# Temporal Variation in the Association between Benzene and Leukemia Mortality

**DOI:** 10.1289/ehp.10841

**Published:** 2008-01-02

**Authors:** David B. Richardson

**Affiliations:** Department of Epidemiology, School of Public Health, University of North Carolina at Chapel Hill, Chapel Hill, North Carolina USA

**Keywords:** benzene, cohort study, leukemia, mortality, Ohio

## Abstract

**Background:**

Benzene is a human carcinogen. Exposure to benzene occurs in occupational and environmental settings.

**Objective:**

I evaluated variation in benzene-related leukemia with age at exposure and time since exposure.

**Methods:**

I evaluated data from a cohort of 1,845 rubber hydrochloride workers. Benzene exposure–leukemia mortality trends were estimated by applying proportional hazards regression methods. Temporal variation in the impact of benzene on leukemia rates was assessed via exposure time windows and fitting of a multistage cancer model.

**Results:**

The association between leukemia mortality and benzene exposures was of greatest magnitude in the 10 years immediately after exposure [relative rate (RR) at 10 ppm-years = 1.19; 95% confidence interval (CI), 1.10–1.29]; the association was of smaller magnitude in the period 10 to < 20 years after exposure (RR at 10 ppm-years = 1.05; 95% CI, 0.97–1.13); and there was no evidence of association ≥ 20 years after exposure. Leukemia was more strongly associated with benzene exposures accrued at ≥ 45 years of age (RR at 10 ppm-years = 1.11; 95% CI, 1.04–1.17) than with exposures accrued at younger ages (RR at 10 ppm-years = 1.01; 95% CI, 0.92–1.09). Jointly, these temporal effects can be efficiently modeled as a multistage process in which benzene exposure affects the penultimate stage in disease induction.

**Conclusions:**

Further attention should be given to evaluating the susceptibility of older workers to benzene-induced leukemia.

In 1982 the International Agency for Research on Cancer (IARC) concluded there was sufficient evidence that benzene is carcinogenic to humans, with evidence predominantly related to associations between benzene and development of acute non-lymphocytic leukemia (IARC 1982). Subsequent epidemiologic studies have supported that conclusion (Hayes et al. 1997; Rinsky et al. 1987; Wong 1987; Yin et al. 1996). In addition, molecular and cytogenic studies provide evidence of induction of chromosomal alterations by benzene that is likely to play a role in leukemogenesis (Smith and Zhang 1998; Zhang et al. 2007).

Despite its status as a recognized leukemogen, benzene exposure is common (IARC 1987). Benzene is an important raw material for the chemical industry and an occasional industrial solvent, as well as a component of gasoline (Hricko 1994). Smokers commonly experience protracted inhalation exposures to benzene as a component of cigarette smoke (Wallace et al. 1987). In addition, environmental exposures to benzene arise from sources such as gasoline vapor emissions and auto exhaust (Wallace 1996). Consequently, the identification of a factor that influences a person’s susceptibility to benzene-induced leukemia has important public health implications, as does understanding the evolution over time of leukemia rates after benzene exposure.

Multistage theories of carcinogenesis predict that a person’s susceptibility to benzene-induced leukemia will depend upon the age at which exposure occurs, as the probability of transition through the stage (or stages) of the disease process unaffected by benzene exposure are assumed to be age dependent (Thomas 1988). Moreover, age-related physiologic changes might lead to changes in susceptibility to benzene’s carcinogenic effects via changes in benzene uptake and its metabolism (Kim et al. 2006). Despite its plausibility as an effect measure modifier, the epidemiologic literature to date provides minimal information about whether susceptibility to benzene-induced leukemia varies with age at exposure.

Multistage cancer models also predict that effect of an increment of exposure on cancer risk may vary with time since exposure. Whereas some investigators have found that a simple metric of cumulative exposure adequately characterizes the exposure time–response relationship (Crump 1994, 1996), others have reported evidence of substantial variation in the impact of benzene exposure on leukemia risk with time since exposure (Finkelstein 2000; Hayes et al. 1997; Silver et al. 2002).

The analyses reported in the present article examine age at exposure and time since exposure as modifiers of the association between the leukemia mortality and occupational benzene exposure in a cohort of rubber hydrochloride workers. Previous analyses of these data have been used by the U.S. Occupational Safety and Health Administration (OSHA) to support the current permissible exposure limit for benzene in the workplace and by the U.S. Environmental Protection Agency (EPA) as the basis for risk estimates for inhaled benzene (OSHA 1987; U.S. EPA 1985). The objective of these analyses was to use exposure time windows and a multistage model to evaluate temporal modifiers of the impact of benzene on leukemia rates.

## Materials and Methods

This study is based upon the experience of workers employed in the manufacture of a natural rubber film (rubber hydrochloride) at two locations in Ohio. Natural rubber was dissolved in benzene and spread over a conveyer; the benzene was evaporated and recovered while the rubber film was stripped from the conveyor (Rinsky et al. 1987). Production at the first location commenced in 1939 and ceased in 1976; production at the second location began around 1937 and continued until 1965. All nonsalaried workers employed in a rubber hydrochloride department between 1 January 1940 and 31 December 1965 were included in these analyses.

Vital status was ascertained through 31 December 1996 via records of the Social Security Administration, Ohio Bureau of Motor Vehicles, and the National Death Index. If there was no death indication for a worker then they were assumed to be alive as of 31 December 1996. Information was obtained on underlying cause of death for deceased workers, coded according to the revision of the *International Classification of Diseases* (ICD) in effect at the time of death. These analyses focus on leukemia {ICD-6 and ICD-7 code 204 [World Health Organization (WHO) 1948, 1957], ICD-8 codes 204-207 [U.S. Public Health Service 1968], ICD-9 codes 204-208 [WHO 1978]}.

The exposure of interest was defined as cumulative benzene exposure, expressed in parts per million-year (ppm-year). Annual exposure rate estimates by plant, department, and job were developed by Rinsky et al. (2002, 1987) based on available air sampling data. Utterbach and Rinsky (1995) have reviewed the methods employed in this assessment of benzene exposure among rubber hydrochloride workers. The U.S. National Institute for Occupational Safety and Health provided a file that contained a plant, department, and job code, and start and finish dates, for each job held by each worker. Using this information, benzene exposure histories were computed for each worker as the product of the length of employment in each job in a year by the estimated benzene exposure rate for that job.

### Statistical methods

Cox proportional hazards regression models were fitted to these data via the statistical program PECAN, with attained age as the primary time scale (Preston et al. 1993). Model covariates included a categorical indicator of birth cohort (classified as born before 1905classified as born before 1905 to < 1910 to < 1910 to < 1915 to < 1915 to < 1920, or after 1920), a binary indicator of sex, and a binary indicator of employment status (active employment status began when a person started employment and ended 1 week after the end of employment in order to allow for inaccuracies in personnel records regarding the day last employed) (Arrighi and Hertz-Picciotto 1994; Steenland and Stayner 1991; Steenland et al. 1996). The majority (99%) of workers of known race in this cohort was white, and no deaths due to leukemia were observed among nonwhite workers; therefore, race was not included as a covariate in these analyses. In analyses of cumulative exposure (expressed in 10-ppm-year increments) log-linear regression models were fitted, providing an estimate of the log relative rate per 10 ppm-years; we report the anti-log of this estimate and discuss it as an estimate of the relative rate at 10 ppm-years. Ninety-five percent confidence intervals (CIs) were estimated via the likelihood method.

Cumulative exposure was treated as a time-varying explanatory variable that described the benzene exposures accrued prior to a person’s entry into a risk set in the Cox regression analysis. The model with a single parameter for cumulative benzene exposure implies that the magnitude of the hazard ratio does not depend on when exposures occurred. Exposure time window analyses were conducted to assess whether the relationship between disease risk and benzene exposure depends on when exposures occurred (Checkoway et al. 1990; Richardson and Ashmore 2005; Thomas 1988). A model with three exposure time windows, defined *a priori*, described the association between leukemia rates and exposures accrued in the periods < 10 years, 10 to < 20 years, and ≥ 20 years prior to a person’s entry into a risk set in the regression analysis (Rothman 1981). To assess variation in exposure effects with age at exposure, metrics of cumulative exposures accrued at < 45 and ≥ 45 years of age were examined (Richardson and Wing 1998). Each model was compared with a standard model of lifetime cumulative exposure by means of a likelihood ratio test (LRT); the difference between model deviances, described as an LRT statistic, can be interpreted using a chi-square distribution with degrees of freedom (df) equal to the difference in the numbers of model parameters.

Multistage models of carcinogenesis, of which the best known is the Armitage–Doll model, involve the mathematic expression of hypotheses about the process of carcinogenesis (Armitage and Doll 1954). Central to the Armitage–Doll model is the concept that cancer arises as the result of a single cell undergoing a series of transformations. The model predicts that cancer incidence, *I*, will increase as an integer power of attained age, *a*, with the integer, depending on the number of stages, *k*, required for cancer induction. Specifically, the model posits the relationship *I = ca^k–1^*, where *c* is a constant that is proportional to the product of the transition rates. When considering the effect of an environmental carcinogen, the transition rate from one rate-limiting step to the next is often assumed to be affected in a linear fashion by exposure. If exposure influences the transition rate for a single stage, *j* < *k*, this implies a linear relative rate model of the form RR (relative rate) = 1 + δ*_j,k_Z*, where *Z* is a weighted cumulative exposure metric calculated for each person (Thomas 1988; Whittemore 1977). Specifically, if *a* denotes the attained age of members of a risk set enumerated for a Cox regression analysis, and *a*_0_ is the age at which an increment of exposure occurs, then the weight assigned to that exposure increment is given by the expression, *w*(*a*_0_) = (1 ÷ *a^k^*^–1^)*a*_0_*^j^*^–1^ (*a*– *a*_0_)*^k^*^–^*^j^*^–1^. The weighted cumulative exposure metric *Z* represents the sum of weighted exposure increments accrued through age *a*.

Leukemia incidence rates increase approximately as a function of age to the fourth power, suggesting a process of carcinogenesis that involves five stages (Little et al. 1992; Ries et al. 2003). Therefore, a disease process that involves five stages was posited (i.e., *k* = 5) and weighted cumulative exposure metrics for each integer value of *j < k* were calculated. Relationships between leukemia mortality and these weighted cumulative exposure metrics were evaluated, and fitted regression models were compared with reference to residual model deviance (−2 log likelihood). Alternative models with fewer than five stages and those with more than five stages were also evaluated. Regression analyses were conducted via the log-linear rate model as well as via the linear relative rate model.

## Results

Table 1 shows the distribution of major characteristics among cases and noncases in the study cohort. A single leukemia death was observed among the females in the study cohort. Over one-third of the leukemia cases were ascertained among workers born before 1905, whereas nearly 60% of the noncases were born in the period 1920 or later. Leukemia cases were employed for a longer average duration than noncases, tended to start employment at older ages than noncases, and accrued higher average cumulative benzene exposures (144 ppm-years) than noncases (34 ppm-years). Two percent of the workers were hired before 1940, 19% were hired in the period 1940–1944, and the remainder were hired in 1945–1975.

Table 2 reports estimated RRs for categories of benzene exposure. The rate ratio for the contrast drawn between the categories 1 to < 50 ppm-years and < 1 ppm-year was below unity (Table 2). When considering contrasts drawn between 50 to < 250, 250 to < 500, and ≥ 500 ppm-years and < 1 ppm-year, the rate ratios were greater than unity and increased in magnitude with increasing cumulative exposure level, although the associated 95% CIs were relatively wide for each exposure category, reflecting the small numbers of leukemia cases observed within each category.

There was a positive trend in the leukemia mortality rate with cumulative benzene exposure (Table 3). Table 3 also describes the association between leukemia and cumulative benzene exposure accrued in the periods < 10 years, 10 to < 20 years, and ≥ 20 years prior. The largest magnitude of association was observed for benzene exposures accrued in the period < 10 years prior, whereas exposures received 10 to < 20 years previously exhibited a smaller, positive association with leukemia, and benzene exposures received ≥ 20 years prior showed no association with leukemia. A model with three exposure time windows provided a substantially better fit to these data than a lifetime cumulative exposure model (LRT = 13.2, 2 df, *p*-value = 0.001).

Table 4 reports the association between cumulative benzene exposures accrued at younger (< 45 years) and older (≥ 45 years) ages and leukemia in the periods < 10 years, 10 to < 20 years, and ≥ 20 years after exposure. When considering benzene exposures accrued at ≥ 45 years of age, there was a positive association with leukemia mortality in the period shortly after exposure (< 10 years after exposure); there was minimal evidence of association within the period ≥ 10 years after exposure. Benzene exposures accrued at younger ages exhibited little evidence of association with leukemia. The fit of this model with exposure time windows defined jointly by age at exposure and time since exposure was substantially better than the fit of a model for lifetime cumulative exposure (LRT = 16.9, 5 df, *p*-value = 0.005). Table 4 also reports estimates of the association between cumulative benzene exposures accrued at younger (< 45 years) and older (≥ 45 years) ages and leukemia, summarized over all periods of time since exposure. A model that included separate terms for two age-at-exposure time windows provided a slightly better fit to these data than the simpler, nested model that included a single parameter for cumulative benzene exposure accrued at all ages (LRT = 3.3, 1 df, *p*-value = 0.071).

The results reported in Tables 3 and 4 are minimally impacted by inclusion of birth cohort, sex, or employment status as covariates; none of the parameter estimates on which the reported effect measures were based changed by > 10% on exclusion of these covariates. The linear relative rate model provided an equivalent fit to these data for analyses of lifetime cumulative exposure; however, the log-linear model fitted these data better for the exposure time window analyses. The cut point defining younger versus older age at exposure was chosen to broadly partition the ages at which exposures occur; there was minimal impact on relative rate estimates of selecting alternative cut points of 40 years or 50 years (results not shown).

**Figure 1:**
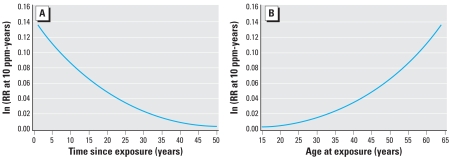
(*A*) Fitted time-varying exposure weighting function. Log relative rate (RR) of leukemia per 10 ppm-year benzene exposure by time since exposure for a person 65 years of age, rubber hydrochloride workers, Ohio, 1940–1996. (*B*). Fitted time-varying exposure weighting function. Log relative rate (RR) of leukemia per 10 ppm-year benzene exposure by age at exposure for a person 65 years of age, rubber hydrochloride workers, Ohio, 1940–1996.

In contrast to the exposure time window analyses presented above, which impose a piecewise constant model to describe temporal variation in exposure effects, the Armitage–Doll model implies a smooth time-varying exposure weighting function that jointly describes age at exposure and latency effects. Residual model deviances were compared for models in which benzene exposure acted upon the first, second, third, or fourth stage of a five-stage disease process (Table 5). A model under which the transition rate for the fourth stage was affected by benzene exposure resulted in the lowest residual deviance and therefore provided the best fit to these data. Figure 1A illustrates how the estimated effect of benzene exposure varies with time since exposure; the figure illustrates the natural log of the estimated relative rate of leukemia per 10 ppm-years for those 65 years of age (i.e., typical of the ages at which leukemia deaths occurred in this population). Consistent with observations from our exposure time window analyses, the modeled effect was largest for exposures that occurred in the prior decade and diminished rapidly with time since exposure. Figure 1B illustrates how the estimated effect of benzene exposure varies with age at exposure. As observed via time window analyses, the exposure effect was much smaller for exposures accrued prior to 45 years of age; the estimated effect of benzene exposure increased with age at exposure > 45 years of age. Multistage models were also fitted using a linear relative rate model; a model in which the transition rate for the penultimate stage was affected by benzene exposure provided the best fit to these data (Table 5). Evaluation of alternative models with as few as three stages, or as many as 15 stages, led to similar conclusions (see Supplemental Material online at http://www.ehponline.org/docs/2008/10841/suppl.pdf); in all such models the best-fitting model is one in which benzene exposure acts at the penultimate stage.

## Discussion

In the United States, the OSHA standard for benzene exposure is 1 ppm. The analyses in the present article suggest that accrual of benzene exposure at that level for a decade implies a modest increase in the relative rate of leukemia mortality, with the magnitude of the excess relative rate diminishing with time since exposure (Table 3). Because leukemia is a rare disease, this means that if a person is exposed to 1 ppm of benzene for a decade, it is still unlikely that they will develop leukemia. To understand the impact of benzene exposure on leukemia risk at a population level, however, the magnitude of the dose–response association and its variation over time must be accurately characterized. In this study population, the effect of benzene exposure on leukemia did not appear to persist indefinitely, but rather diminished with time since exposure. Of course, caution is warranted in drawing conclusions from an historical cohort study of a population in which working conditions differed substantially from those typical of contemporary work settings in the United States. Nonetheless, the findings of this historical cohort of U.S. workers may have substantial relevance for contemporary workers, both in the United States and abroad.

In prior analyses of this cohort, Crump (1994, 1996) investigated the hypothesis that the effect of benzene on leukemia risk diminishes with time since exposure by applying a set of time-dependent exposure weights with values informed *a priori* by latency patterns for leukemia after radiotherapy for ankylosing spondylitis. Crump reported that analyses using a simple metric of cumulative exposure fitted these data better than analyses using those exposure weights (Crump 1996). In the present paper, rather than assigning a set of exposure weights based on patterns observed in a study of radiation exposure effects, the method of exposure time–window analysis was used. The overall association between cumulative exposure and leukemia mortality (RR at 10 ppm-years = 1.05) is nearly identical to the estimate derived by Rinsky et al. (2002) via a log-linear Cox regression model; the evidence of heterogeneity of benzene exposure effects with time since exposure is consistent with previous observations reported by Silver et al. (2002) and Finkelstein (2000).

These findings suggest that the effect of benzene on leukemia mortality is jointly characterized as an effect of age at exposure and time since exposure. The temporal pattern is consistent with a multistage cancer model with benzene affecting a late stage in the induction of leukemia; the relative rate of leukemia per unit exposure increases with age at exposure and decreases with time since exposure (Thomas 1988). This conclusion is supported by analyses that involve fitting weighting expressions implied by the Armitage–Doll model. These weighted exposure metrics were evaluated via fittings of standard log-linear models as well as via fittings of linear relative rate models [the latter being the model form implied by the work of Whittemore (1977), whereas the former approach was consistent with the model form used in the exposure time–window analyses]. In these analyses a model with five stages was posited. Armitage and Doll intentionally used the word “stage” rather than mutation to allow for the possibility of nonmutational events leading to cancer induction (Doll 2004). They correctly maintained that the application of multistage models for cancer risk estimation offers a heuristic tool that allows an investigator to explore potentially complex dose-time–response patterns by imposing some relatively minor constraints based on biological expectations about the disease process. Although mutational events are clearly central to carcinogenesis, useful insights from these models may be obtained even if carcinogenesis is viewed more generally as resulting from a series of rate-limiting pathogenic events, with exposure influencing one or more transition rates (Hanahan and Weinberg 2000; Morrison 1979).

The validity of these findings depends, in part, on the validity of the benzene exposure estimates derived for this cohort. To the extent that the exposure measurement error conforms to a classical model, attenuation of the dose response would be expected. However, non-random measurement errors could lead to bias away from the null. Estimates of these historical benzene exposures used air monitoring results, which were relatively sparse for the early years of operation (Utterback and Rinsky 1995; Williams and Paustenbach 2003). In theory, temporal variation in the magnitude of a benzene–leukemia association (e.g., diminished evidence of association with increasing time since exposure) could reflect increasing exposure misclassification for benzene exposure estimates for periods of employment further in the past. While it is difficult to assess such concerns, the observation in this cohort that the benzene–leukemia association diminished with time since exposure is consistent with patterns observed in other populations of benzene-exposed workers (Glass et al. 2004; Hayes et al. 1996), suggesting that the temporal patterns in this cohort are not simply an artifact of errors in exposure estimates.

Although the fitted models include a relatively small number of covariates, concerns about bias because of residual confounding are tempered by the fact that there are few leukemogens that are plausible strong confounders of the association under study. Cigarette smoking is a nonoccupational source of benzene exposure and could, in theory, confound our estimates of association between occupational benzene exposure and leukemia. However, given the relatively small magnitude of association between smoking and leukemia mortality, high levels of correlation between occupational benzene exposure and smoking would be necessary to account for even modest dose–response trends for leukemia (Axelson and Steenland 1988; Siemiatycki et al. 1988).

The analyses in this article examined the broad category of all leukemia deaths. It is reasonable to posit that associations may vary in magnitude and temporal pattern by disease subtype. Although evaluation of heterogeneity in exposure–response analyses for different subtypes of leukemia is of interest because of small numbers of leukemia cases and the sparse information available from the death certificates, subtype-specific exposure–response analyses were not conducted. In addition, the use of mortality data in these analyses does not allow assessment of whether benzene exposure influences disease prognosis or incidence; therefore, it is possible that benzene exposures accrued proximate to death could influence mortality rates by reducing survival time rather than by increasing incidence rates. The relatively small number of leukemia deaths also suggests that model results are relatively sensitive to small changes in distribution of events; adding or subtracting a single case in the highest exposure category could lead to a substantial change in the estimates of the association between cumulative exposure and leukemia mortality. Last, the Armitage–Doll model, while often illustrated using mortality data (Armitage and Doll 1954), is posited as a model of disease incidence; it is likely that the conclusions obtained in these analyses would differ from those obtained via analyses of incidence data.

Since 1987, the Chinese Academy of Preventive Medicine has collaborated with the U.S. National Cancer Institute on a large-scale study of cancer among Chinese workers exposed to benzene (NCI-CAPM study) (Hayes et al. 1997). Although the NCI-CAPM study encompasses more leukemia cases than in this rubber hydrochloride cohort study, several concerns have been raised about the validity of the exposure estimates used in the previously reported analyses of the NCI-CAPM study (Hayes et al. 2001). Therefore, the rubber hydrochloride cohort examined in this article remains one of the important epidemiologic resources for benzene risk assessment.

The findings illustrate the importance of attention to dynamic changes in exposure–response patterns with temporal factors such as time since exposure and age at exposure. Failure to account for variation with time since exposure in the effect of an increment of benzene exposure on the relative rate of leukemia may lead to underestimation of the excess rate of leukemia in some risk periods (and overestimation of the excess rate of leukemia in other risk periods). In these analyses, the effect of an increment of benzene exposure on leukemia mortality appears promptly, diminishes with time since exposure, and is of greater magnitude for workers exposed at older ages than for those exposed at younger ages. These temporal patterns of association are consistent with a late-stage carcinogen and suggest that occupational protection efforts give particular consideration to the risks of benzene-induced leukemia faced by older workers. Further attention should be given to assessment of age at exposure in other benzene-exposed populations, specifically to the potentially greater susceptibility of older workers to benzene-induced leukemia.

## Figures and Tables

**Table 1:** Characteristics [*n* (%)] of cohort of 1,845 rubber hydrochloride workers stratified by leukemia case status, Ohio, 1940–1996.

Characteristic	Cases (*N* = 17)	Noncases (*N* = 1,828)
Sex		
Male	16 (94)	1,705 (93)
Female	1 (6)	123 (7)
Birth cohort		
< 1905	6 (35)	230 (13)
1905 – <1910	2 (12)	131 (7)
1910 – <1915	3 (18)	193 (11)
1915 – <1920	3 (18)	226 (12)
< 1920	3 (18)	1,048 (57)
Employment status		
Employed	2 (12)	10 (1)
Terminated	15 (88)	1,818 (99)
Age at entry (years, mean ± SD)	41 ± 11	32 ± 11
Age at exit (years, mean ± SD)	62 ± 17	67 ± 12
Duration of employment (years, mean ± SD)	7 ± 8	4 ± 7
Cumulative exposure (ppm-years, mean ± SD)	144 ± 207	34 ± 91

**Table 2:** Estimated association between cumulative exposure to benzene and leukemia mortality among rubber hydrochloride workers, Ohio, 1940–1996.

	Cumulative exposure to benzene (ppm-years)				
	< 1	1 to < 50	50 to < 250	250–500	≥ 500
RR (95% CI)	1	0.8 (0.2–3.2)	2.5 (0.6–10.2)	10.5 (2.3–46.6)	13.9 (0.7–116.1)
Deaths (no.)	5	3	4	4	1

**Table 3:** Estimated relative rates (and associated 95% CIs) for leukemia mortality expressed as a trend with benzene exposure (10 ppm-years) and within time windows defined by time since exposure.

	RR at 10 ppm-years (95% CI)
Cumulative exposure	1.05 (1.02–1.08)
Time since exposure	
< 10 years prior	1.19 (1.10–1.29)
10 to < 20 years prior	1.05 (0.97–1.13)
≥ 20 years prior	1.00 (0.90–1.05)
Test of heterogeneity	
LRT, 2 df^a^	13.1
*p*-Value	0.001

a LRT comparing a model with terms for three exposure time windows to a model with one term for lifetime cumulative exposure.

**Table 4:** Estimated association between leukemia mortality and cumulative exposure to benzene in exposure time windows cross-classified by age at exposure and time since exposure.

	RR at 10 ppm-years (95% CI)	
	Accrued at < 45 years of age	Accrued at ≥ 45 years of age
Cumulative exposure	1.01 (0.92–1.09)	1.11 (1.04–1.17)
Time since exposure		
< 10 years prior	0.78 (ND–1.23)	1.22 (1.11–1.32)
10 to < 20 years prior	1.05 (0.89–1.22)	1.03 (0.92–1.13)
≥ 20 years prior	1.01 (0.90–1.09)	0.93 (0.55–1.10)

ND, not determined (the 95% confidence bound was not determined via the likelihood method). LRT comparing model with six exposure time windows to the cumulative exposure model = 16.9, 5 df, *p*-value = 0.005.

**Table 5:** Residual deviances from fitting of log-linear and linear RR regression models.

Stage affected by benzene (*j*)	Log-linear rate model	Linear RR model
1	211.23	209.5
2	209.76	206.9
3	204.74	203.4
4	193.60	200.1

Comparison of models in which a cumulative weighted benzene exposure metric was derived via a multistage model with five stages (i.e., *k* = 5), assuming a single stage, *j*, was affected by benzene exposure.

## References

[b1-ehp0116-000370] Armitage P, Doll R (1954). The age distribution of cancer and a multistage theory of carcinogenesis. Br J Cancer.

[b2-ehp0116-000370] Arrighi HM, Hertz-Picciotto I (1994). The evolving concept of the healthy worker survivor effect. Epidemiology.

[b3-ehp0116-000370] Axelson O, Steenland K (1988). Indirect methods of assessing the effects of tobacco use in occupational studies. Am J Ind Med.

[b4-ehp0116-000370] Checkoway H, Pearce N, Hickey JL, Dement JM (1990). Latency analysis in occupational epidemiology. Arch Environ Health.

[b5-ehp0116-000370] Crump KS (1994). Risk of benzene-induced leukemia: a sensitivity analysis of the pliofilm cohort with additional follow-up and new exposure estimates. J Toxicol Environ Health.

[b6-ehp0116-000370] Crump KS (1996). Risk of benzene-induced leukemia predicted from the Pliofilm cohort. Environ Health Perspect.

[b7-ehp0116-000370] Doll R (2004). Commentary: The age distribution of cancer and a multistage theory of carcinogenesis. Int J Epidemiol.

[b8-ehp0116-000370] Finkelstein MM (2000). Leukemia after exposure to benzene: temporal trends and implications for standards. Am J Ind Med.

[b9-ehp0116-000370] Glass DC, Sim MR, Fritschi L, Gray CN, Jolley DJ, Gibbons C (2004). Leukemia risk and relevant benzene exposure period—re: follow-up time on risk estimates. Comment on Am J Ind Med 42:481–489, 2002. Am J Ind Med.

[b10-ehp0116-000370] Hanahan D, Weinberg RA (2000). The hallmarks of cancer. Cell.

[b11-ehp0116-000370] Hayes RB, Yin SN, Dosemeci M, Li GL, Wacholder S, Chow WH (1996). Mortality among benzene-exposed workers in China. Environ Health Perspect.

[b12-ehp0116-000370] Hayes RB, Yin SN, Dosemeci M, Li GL, Wacholder S, Travis LB (1997). Benzene and the dose-related incidence of hematologic neoplasms in China. Chinese Academy of Preventive Medicine—National Cancer Institute Benzene Study Group. J Natl Cancer Inst.

[b13-ehp0116-000370] Hayes RB, Songnian Y, Dosemeci M, Linet M (2001). Benzene and lymphohematopoietic malignancies in humans. Am J Ind Med.

[b14-ehp0116-000370] Hricko A (1994). Rings of controversy around benzene. Environ Health Perspect.

[b15-ehp0116-000370] IARC (International Agency for Research on Cancer) (1982). Benzene. In: Some Industrial Chemicals and Dyestuffs. IARC Monogr Eval Carcinog Risk Chem Hum.

[b16-ehp0116-000370] IARC (International Agency for Research on Cancer) (1987). Overall Evaluations of Carcinogenicity: An Updating of IARC Monographs 1 to 42. Monogr Eval Carcinog Risk Chem Hum.

[b17-ehp0116-000370] Kim S, Vermeulen R, Waidyanatha S, Johnson BA, Lan Q, Smith MT (2006). Modeling human metabolism of benzene following occupational and environmental exposures. Cancer Epidemiol Biomarkers Prev.

[b18-ehp0116-000370] Little MP, Hawkins MM, Charles MW, Hildreth NG (1992). Fitting the Armitage-Doll model to radiation-exposed cohorts and implications for population cancer risks. Radiat Res.

[b19-ehp0116-000370] Morrison AS (1979). Sequential pathogenic components of rates. Am J Epidemiol.

[b20-ehp0116-000370] OSHA (1987). Occupational expsoure to benzene; final rule. Fed Reg.

[b21-ehp0116-000370] Preston DL, Lubin JH, Pierce DA, McConney ME (1993). Epicure: User’s Guide.

[b22-ehp0116-000370] Richardson D, Ashmore JP (2005). Investigating Time Patterns of Variation in Radiation-Cancer Associations. Occup Environ Med.

[b23-ehp0116-000370] Richardson DB, Wing S (1998). Methods for investigating age differences in the effects of prolonged exposures. Am J Ind Med.

[b24-ehp0116-000370] Ries L, Eisner M, Kosary C (2003). SEER Cancer Statistics Review, 1975–2000.

[b25-ehp0116-000370] Rinsky RA, Hornung RW, Silver SR, Tseng CY (2002). Benzene exposure and hematopoietic mortality: a long-term epidemiologic risk assessment. Am J Ind Med.

[b26-ehp0116-000370] Rinsky RA, Smith AB, Hornung R, Filloon TG, Young RJ, Okun AH (1987). Benzene and leukemia: an epidemiologic risk assessment. N Engl J Med.

[b27-ehp0116-000370] Rothman KJ (1981). Induction and latent periods. Am J Epidemiol.

[b28-ehp0116-000370] Siemiatycki J, Wacholder S, Dewar R, Wald L, Begin D, Richardson L (1988). Smoking and degree of occupational exposure: are internal analyses in cohort studies likely to be confounded by smoking status?. Am J Ind Med.

[b29-ehp0116-000370] Silver SR, Rinsky R, Cooper SP, Hornung RW, Lai D (2002). Effect of Follow-up time on risk estimates: a longitudinal examination of the relative risks of leukemia and multiple myeloma in a rubber hydrochlorine cohort. Am J Ind Med.

[b30-ehp0116-000370] Smith MT, Zhang L (1998). Biomarkers of leukemia risk: benzene as a model. Environ Health Perspect.

[b31-ehp0116-000370] Steenland K, Deddens J, Salvan A, Stayner L (1996). Negative bias in exposure-response trends in occupational studies: modeling the healthy workers survivor effect. Am J Epidemiol.

[b32-ehp0116-000370] Steenland K, Stayner L (1991). The importance of employment status in occupational cohort mortality studies. Epidemiology.

[b33-ehp0116-000370] Thomas DC (1988). Models for exposure-time-response relationships with applications to cancer epidemiology. Annu Rev Public Health.

[b34-ehp0116-000370] U.S. EPA (1985). Interim Quantitative Cancer Unit Risk Estimates Due to Inhalation of Benzene.

[b35-ehp0116-000370] U.S. Public Health Service (1968). International Classification of Diseases Adapted for Use in the United States, Eighth Revision. U.S. Public Health Service Publ. no. 1693.

[b36-ehp0116-000370] Utterback DF, Rinsky RA (1995). Benzene exposure assessment in rubber hydrochloride workers: a critical evaluation of previous estimates. Am J Ind Med.

[b37-ehp0116-000370] Wallace L (1996). Environmental exposure to benzene: an update. Environ Health Perspect.

[b38-ehp0116-000370] Wallace L, Pellizzari E, Hartwell TD, Perritt R, Ziegenfus R (1987). Exposures to benzene and other volatile compounds from active and passive smoking. Arch Environ Health.

[b39-ehp0116-000370] Whittemore AS (1977). The age distribution of human cancer for carcinogenic exposures of varying intensity. Am J Epidemiol.

[b40-ehp0116-000370] WHO (1948). International Statistical Classification of Diseases, Injuries, and Causes of Death. Sixth Revision.

[b41-ehp0116-000370] WHO (1957). International Statistical Classification of Diseases. Seventh Revision.

[b42-ehp0116-000370] WHO (1978). International Classification of Diseases. Ninth Revision.

[b43-ehp0116-000370] Williams PR, Paustenbach DJ (2003). Reconstruction of benzene exposure for the Pliofilm cohort (1936–1976) using Monte Carlo techniques. J Toxicol Environ Health A.

[b44-ehp0116-000370] Wong O (1987). An industry wide mortality study of chemical workers occupationally exposed to benzene. II. Dose response analyses. Br J Ind Med.

[b45-ehp0116-000370] Yin SN, Hayes RB, Linet MS, Li GL, Dosemeci M, Travis LB (1996). An expanded cohort study of cancer among benzene-exposed workers in China. Benzene Study Group. Environ Health Perspect.

[b46-ehp0116-000370] Zhang L, Rothman N, Li G, Guo W, Yang W, Hubbard AE (2007). Aberrations in chromosomes associated with lymphoma and therapy-related leukemia in benzene-exposed workers. Environ Mol Mutagen.

